# Gleanings from Our Exchanges

**Published:** 1874-03-15

**Authors:** 


A CAUSE OF NIGHTMARE.—Many children, and some grownup folks, suffer terribly from nightmare. A frequent and hitherto unknown cause has been pointed out by Dr. Warrington Howard. He found, in some cases, the attack greatly aggravated by *enlarged tonsils*, and entirely dispersed when these were removed.—*Phil. Med. Rep.*

Spontaneous Expulsion of the Uterus.—Dr. Martin, of Toulouse, relates (in *L'Union Medicale* No. 79, 1873) a rare case of spontaneous expulsion of the uterus. A lady aged thirty-five years, having given birth to a child sixteen years previously, had been suffering for some time with ulceration of the os uteri, rapidly progressing, and attended with copious hæmorrhage and great pains. Last 20th of June, the patient, with much straining, though without flooding, had passed per vaginum, a solid body, which subsequent examination *proved to be the body of the uterus.* A vaginal exploration, on account of danger of hæmorrhage, was postponed until the 16th, though in the meantime there was no bleeding; while the involuntary discharge of urine raised a suspicion of vesico-vaginal fistula. On the 20th, the comparatively well being gave way to disease, soon diagnosed as peritonitis, which ended fatally June 23d. An autopsy on the following day confirmed the diagnosis, and showed the true pelvis filled with pus. No trace of the womb was discovered; the round and broad ligaments were destroyed; one ovary enlarged, and the other hypertrophied. The bladder was intact, but right ureter disorganized, whence the flow of urine. The author is of the opinion that, without the fatal peritonitis, a continuation of life would not have been impossible.

---

Paracentesis Thoracis (*The Practitioner*, December, 1873).—Dr. Sydney Ringer publishes his notes on five cases of paracentesis thoracis, showing, by them, how slight a disturbance this operation causes, and what immense relief it affords; showing, also, that the operation may be usefully employed in the febrile and non-febrile stages of pleurisy with effusion, and that during fever the fluid may be withdrawn by the aspirator, and not accumulate again. In some cases of empyema, it is sufficient to withdraw part of the fluid by the aspirator. The rest may disappear; so that it is not always necessary to lay open the chest in order that the pus may drain entirely away. In severe empyema, the temperature may be normal, or scarcely at all raised; and in those cases, accompanied by chronic fever, the pus may be perfectly sweet.—*Phila. Medical Times.*

THE nativity of Adam is not a matter of doubt with the Darwinians, who believe him to have been a germ-man.—*Boston Jour. of Chem.*

